# Doxycycline-Mediated Control of Cyclin D2 Overexpression in Human-Induced Pluripotent Stem Cells

**DOI:** 10.3390/ijms25168714

**Published:** 2024-08-09

**Authors:** Aijun Qiao, Yuhua Wei, Yanwen Liu, Asher Kahn-Krell, Lei Ye, Thanh Nguyen, Jianyi Zhang

**Affiliations:** 1 Department of Biomedical Engineering, School of Medicine and School of Engineering, University of Alabama at Birmingham, Birmingham, AL 35294, USA; aqiao@uab.edu (A.Q.); ywei@uabmc.edu (Y.W.); lywxxy@uab.edu (Y.L.); akrell@uab.edu (A.K.-K.); lye@uab.edu (L.Y.); thamnguy@uab.edu (T.N.); 2 Department of Medicine/Cardiovascular Diseases, University of Alabama at Birmingham, Birmingham, AL 35294, USA

**Keywords:** human-induced pluripotent stem cells, cyclin D2, doxycycline-inducible Tet-On transactivator, myocardial regeneration

## Abstract

Previous studies have demonstrated that when the cyclin D2 (CCND2), a cell-cycle regulatory protein, is overexpressed in human-induced pluripotent stem cells (hiPSCs), cardiomyocytes (CMs) differentiated from these CCND2-overexpressing hiPSCs can proliferate after transplantation into infarcted hearts, which significantly improves the cells’ potency for myocardial regeneration. However, persistent CM proliferation could lead to tumor growth or the development of arrhythmogenic complications; thus, the goal of the current study was to generate a line of hiPSCs in which CCND2 overexpression could be tightly controlled. First, we transfected hiPSCs with vectors coding for a doxycycline-inducible Tet-On transactivator and *S. pyogenes* dCas9 fused to the VPR activation domain; then, the same hiPSCs were engineered to express guide RNAs targeting the CCND2 promotor. Thus, treatment with doxycycline (dox) activated dCas9-VPR expression, and the guide RNAs directed dCas9-VPR to the CCND2 promoter, which activated CCND2 expression. Subsequent experiments confirmed that CCND2 expression was dox-dependent in this newly engineered line of hiPSCs (^dox^CCND2-hiPSCs): CCND2 protein was abundantly expressed after 48 h of treatment with dox and declined to near baseline level ~96 h after dox treatment was discontinued.

## 1. Introduction

Because mammalian cardiomyocytes (CMs) undergo cell-cycle arrest during the perinatal period, CMs in adult mammals’ hearts cannot self-replicate; consequently, the damage caused by myocardial injury or disease often leads to adverse remodeling and heart failure [1]. Thus, investigators have attempted to replenish the myocardial scar with functional contractile tissue by injecting human-induced pluripotent stem cell (hiPSC)-derived CMs (hiPSC-CMs) or implanting hiPSC-CM-containing engineered cardiac muscle patches [2,3,4,5]. Both strategies have been associated with improvements in scar size and functional recovery when studied in animal models of myocardial injury, and we have shown that when hiPSCs are transfected with vectors coding for the cell-cycle regulatory protein cyclin D2 (CCND2), the CMs differentiated from these CCND2-overexpressing hiPSCs can proliferate after transplantation, which significantly increases the cells’ potency for myocardial regeneration [6,7].

However, persistent CM proliferation could lead to tumor growth or the development of arrhythmogenic complications; therefore, techniques for controlling the onset and duration of CCND2 overexpression in hiPSC-derived cells may be needed to facilitate the clinical translation of this promising therapeutic strategy. Thus, for the experiments described in this report, we combined two genome-editing techniques, clustered regularly interspaced short palindromic repeats (CRISPRs) and transcription-activator–like effector nucleases (TALENs) [8,9], to generate hiPSCs that overexpress CCND2 only in the presence of doxycycline (dox) [10,11]. Subsequent studies confirmed that CCND2 was robustly overexpressed in dox-inducible CCND2-overexpressing hiPSCs (^dox^CCND2-hiPSCs) after 48 h of dox treatment and declined to near baseline level ~96 h after dox treatment was discontinued.

## 2. Results

### 2.1. Generation of hiPSCs That Express dCas9 Only in the Presence of Dox (^dox^dCas9-hiPSCs)

**Figure 1:**
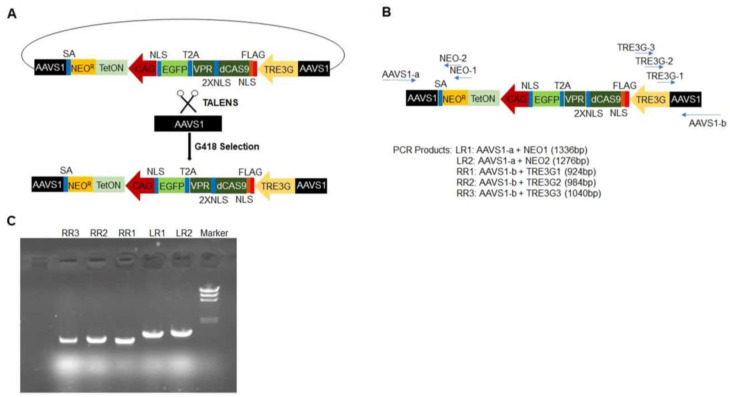
Strategy for generating ^dox^dCas9-hiPSCs. (**A**) The all-in-one cassette used to generate ^dox^dCas9-hiPSCs is displayed as a schematic. It includes a neomycin-resistance sequence (NEO^R^), an rtTA sequence (TetON) driven by the CAG promoter, and a sequence coding for dCas9-VPR linked to EGFP by a self-cleaving T2A peptide, which is driven by the TRE3G promoter. The sequence was inserted into the AAVS1 locus of hiPSCs via TALENS, and successfully transfected cells were collected via treatment with G418 (a neomycin analog) and flow-cytometry selection for EGFP fluorescence (SA: splice acceptor; NLS, 2XNLS, FLAG). (**B**) Genomic DNA primers were generated for two regions spanning the left AAVS1 and NEO^R^ sequences and for three regions spanning the right AAVS1 and TRE3G sequences; then, (**C**) the successful insertion of the all-in-one cassette was confirmed via genomic DNA PCR.

**Figure 2:**
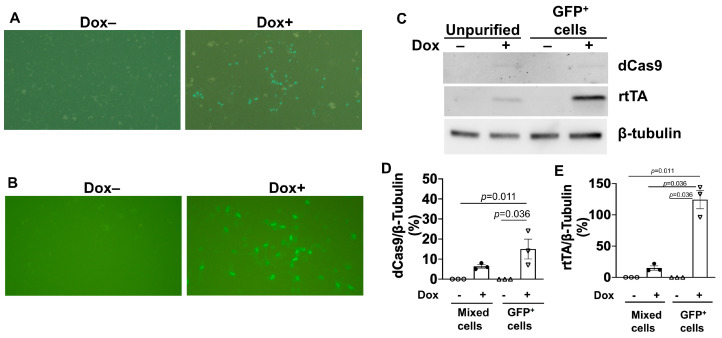
Dox treatment induced GFP expression and upregulated dCas9 and rtTA protein abundance in ^dox^dCas9-hiPSCs. (**A**) GFP fluorescence was visualized in unpurified ^dox^dCas9-hiPSCs (i.e., after G418 selection) that had been treated with or without 5 μg/mL dox (Dox+ or Dox-, respectively) for 48 h. (**B**) GFP fluorescence was visualized in purified ^dox^dCas9-hiPSCs (i.e., after both G418 selection and flow-cytometry sorting for GFP fluorescence) that had been treated with or without dox for 48 h. (**C**) dCas9 and rtTA protein expression levels were evaluated by Western blot analysis in unpurified and purified ^dox^dCas9-hiPSCs after treatment with or without dox for 48 h. Quantification of dCas9 (**D**) and rTTA (**E**) protein expressions after being normalized to β-tubulin protein. Statistical testing: Kruskal-Wallis test, sample size *n* = 3.

^dox^Cas9-hiPSCs were generated by transfecting unmodified hiPSCs with an all-in-one cassette containing a dox-inducible Tet-On transactivator (rtTA) and *S. pyogenes* Cas9 Endonuclease Dead (dCas9), which was fused to the VPR activation domain, a T2A self-cleaving peptide sequence, and Enhanced Green Fluorescent Protein (EGFP); the expression of the complete dCas9-VPR-T2A-EGFP construct was driven by the dox-inducible TREG3 promoter (Figure 1A). The cassette also conferred neomycin (G418) resistance and was inserted into the AAVS1 safe-harbor locus via a TALEN-mediated gene-trap approach. Ten G418-resistant, EGFP-positive clones were selected and genotyped by amplifying the homology arms flanking the neomycin-resistance (left) and TRE3G (right) sequences (Figure 1B). Cells from one of the clones displayed bands that were consistent with the correctly targeted insertion of the AAVS1 alleles (Figure 1C), and when treated with dox, 20% of the cells expressed EGFP (Figure 2A). Dox-treated cells were subsequently purified via flow-cytometry for GFP fluorescence, which increased the proportion of GFP-expressing cells to 100% (Figure 2B) and dramatically increased dCas9 and rtTA protein abundance (Figure 2C–E), but dCas9 protein was not detected in the absence of dox treatment, as reported previously [8]. doxdCas9-hiPSCs were also evaluated via karyotype analysis to confirm the absence of any chromosomal abnormalities.

### 2.2. Generation of hiPSCs That Overexpress CCND2 Only in the Presence of Dox (^dox^CCND2-hiPSCs)

**Figure 3:**
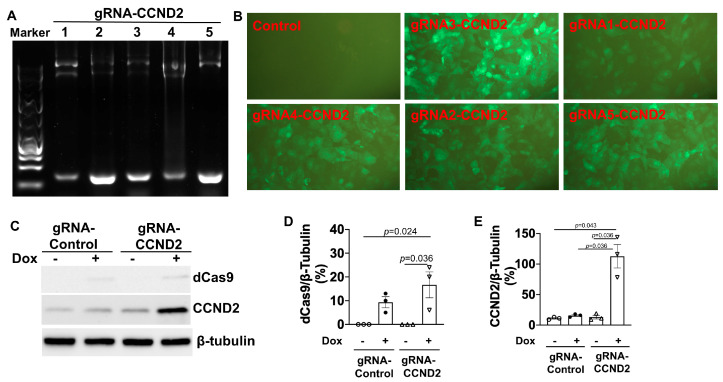
Generation of ^dox^CCND2-hiPSCs. (**A**) Five different CCND2 guide RNA sequences (gRNA-CCND2 numbers 1-5) were validated via PCR analysis with the U6 validation primer and the gRNA reverse primer. (**B**) GFP fluorescence was visualized in ^dox^CCND2-hiPSCs that had been generated via transduction with lentiviruses coding for the CCND2 guide RNAs and in ^dox^dCas9-hiPSCs that had been transduced with a control guide RNA after the cells were treated with dox for 48 h. (**C**) dCas9 and CCND2 protein expression levels were evaluated by Western blot analysis in ^dox^dCas9-hiPSCs that had been transduced with a control guide RNA (gRNA-Control) or gRNA-CCND2 after treatment with (+) or without (-) dox for 48 h. Quantification of dCas9 (**D**) and CCND2 (**E**) protein expressions after being normalized to β-tubulin protein. Statistical testing: Kruskal-Wallis test, sample size *n* = 3.

**Figure 4:**
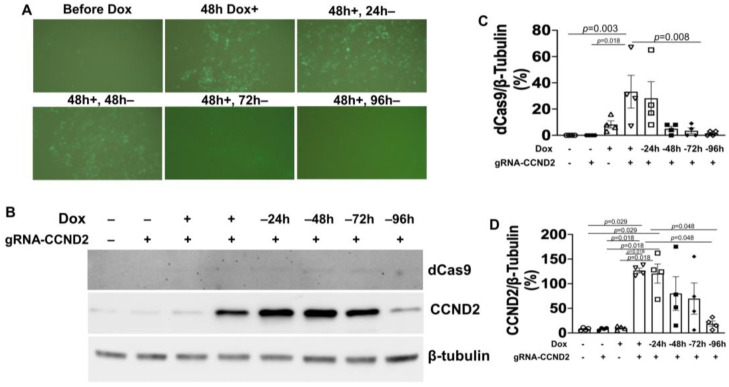
GFP and CCND2 expression in ^dox^CCND2-hiPSCs can be turned on and off by dox treatment and withdrawal. ^dox^CCND2-hiPSCs and ^dox^dCas9-hiPSCs were treated with 5 μg/mL dox for 48 h; then, dox treatment was withdrawn, and the cells were maintained for an additional 96 h. (**A**) GFP fluorescence was visualized in ^dox^CCND2-hiPSCs before dox treatment; after treatment with dox for 48 h (48 h Dox+); and then after the dox-treated cells had been maintained in the absence of dox for 24 (48 h+, 24 h-), 48 (48 h+, 48 h-), 72 (48 h+, 72 h-), and 96 h (48 h+, 96 h-). (**B**) dCas9 and CCND2 protein expression levels were evaluated by Western blot analysis in ^dox^CCND2-hiPSCs in the absence of dox (-) and at the indicated time points after dox treatment and withdrawal. Quantification of dCas9 (**C**) and CCND2 (**D**) protein expressions after being normalized to β-tubulin protein. Statistical testing: Kruskal-Wallis test, sample size *n* = 4.

**Figure 5:**
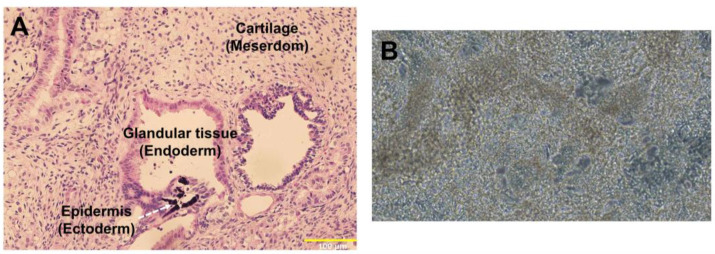
Pluripotency of ^dox^CCND2-hiPSCs. (**A**) ^dox^CCND2-hiPSCs formed a teratoma containing mesodermal (cartilage), ectodermal (epidermis), and endodermal (glandular tissue) cells after implantation into an immunodeficient mouse. (**B**) A representative image (20× magnification) of CMs differentiated from ^dox^CCND2-hiPSCs.

Five gRNAs targeting the CCND2 (gRNA-CCND2) promoter region were designed, linked to a GFP gene expression cassette, and packed into lentivirus vectors. Vector construction was validated via both genomic DNA PCR analysis (Figure 3A) and DNA sequencing, and the efficiency of vector transduction was confirmed by monitoring GFP fluorescence in unmodified hiPSCs that had been treated with the gRNA-CCND2-GFP construct (Figure 3B); then, the same vectors were transduced into ^dox^dCas9-hiPSCs to generate ^dox^CCND2-hiPSCs. Subsequent Western blot analysis confirmed that after 48 h of treatment with dox, CCND2 protein was abundantly expressed in ^dox^CCND2-hiPSCs but not in ^dox^dCas9-hiPSCs that had been transfected with viruses coding for a control gRNA (gRNA-control) sequence (Figure 3C–E). Furthermore, GFP fluorescence dramatically reduced 72 h after the withdrawal of dox treatment (Figure 4A), while both dCas9 and CCND2 protein abundance returned to baseline levels ~96 h after the withdrawal of dox treatment (Figure 4B–D). The ^dox^CCND2-hiPSCs maintained pluripotency as demonstrated in Figure 5.

## 3. Discussion

**Figure 6:**
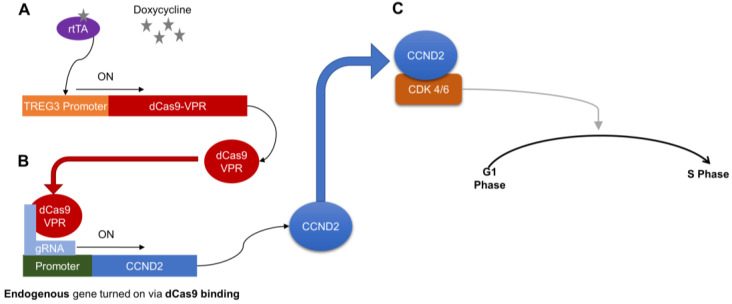
Schematic summary of how dox treatment controls proliferation in CMs differentiated from ^dox^CCND2-hiPSCs. A—Doxycycline induces a conformational change in rtTA, which binds the TREG3 promoter to activate dCas9-VPR expression. B—Guide RNAs (gRNA) direct dCas9-VPR to the CCND2 promoter, which activates CCND2 expression. C—CCND2 binds cyclin-dependent kinases 4/6 (CDK 4/6), which triggers signaling pathways that subsequently activate the G1-S phase transition of the cell cycle.

Previous studies have demonstrated that CCND2 overexpression not only increases the proliferative and regenerative potency of hiPSC-CMs, but can also promote cell-cycle activity in the endogenous CMs of infarcted mouse hearts [6,7,12,13,14,15]. However, the persistent uncontrolled expression of cell-cycle regulatory molecules could lead to the development of tumors, as well as arrhythmogenic complications; for example, when adenovirus coding for microRNA-199a was administered to infarcted pig hearts, CM proliferation increased, and the treatment improved measurements of contractile activity and scar size, but most of the animals died from sudden arrhythmia [16]. Thus, the goal of the current study was to combine the dox-inducible dCas9-VPR system with gRNA targeting the CCND2 promoter to generate a line of hiPSCs in which CCND2 overexpression could be tightly controlled (Figure 6) [11,17]. Importantly, dox treatment does not appear to alter the pluripotency of hiPSCs and may even increase hiPSC survival and self-renewal [8,18].

The AAVS1 locus was chosen as the integration site because previous investigations have demonstrated that insertions in this locus do not alter gene function, and the transgene is consistently expressed for over 50 cell divisions [19]. Dox-induced dCas9-VPR expression also remains stable when the cells are maintained for over 6 months [8], which suggests that the decline in CCND2 expression observed after dox was washed out of the culture medium was caused by an absence of dCas9-VPR activity. However, we have not conclusively determined whether the CCND2-promoter–targeting gRNA expression may also have decayed. Notably, the AAVS1 locus is located within an intron of an active gene (PPP1R12C), so a splicing-acceptor-linked puromycin-resistant gene could be used to exclude untransfected cells and cells in which the transgene was only integrated randomly [11]. However, this strategy would not eliminate cells with both successfully targeted and random integration sites; thus, genome DNA sequencing analysis was performed to ensure that the selected clones contained only correctly targeted insertions.

In conclusion, we have generated a line of hiPSCs in which a dox-inducible dCas9-VPR system drives CCND2 overexpression. CCND2 was abundantly overexpressed in ^dox^CCND2-hiPSCs after 48 h of dox treatment and returned to near-background levels ~96 h after treatment was withdrawn. The ongoing development of this novel hiPSC line may enable the proliferation of hiPSC-derived cells, especially hiPSC-CMs, to be precisely controlled, thereby minimizing the tumorigenic and arrhythmogenic risks associated with persistent CM proliferation.

## 4. Materials and Methods

### 4.1. Plasmids

All plasmids were purchased from Addgene (pT076: catalog number 137880; pZT-AAVS1-L1: catalog number 52637; pZT-AAVS1-R1: catalog number 52638; pMD2.G: catalog number 12259; psPAX2: catalog number 12260; and pLKO5.sgRNA.EFS.GFP: catalog number 57822; Watertown, MA, USA).

### 4.2. Generation of Dox-Inducible dCas9-VPR hiPSCs

An established hiPSC line [20] was grown in mTeSR1 medium (Stem Cell Technologies 05850; Vancouver, BC, Canada) on Geltrex-coated (Life Technologies A1413301; Waltham, MA, USA) plates and repeatedly tested to confirm the absence of mycoplasma contamination (Lonza MycoAlert LT07-418). To integrate the Tet-ON and dCas9-VPR constructs into the AAVS1 locus, 2.5 × 10^6^ cells were co-transfected with 10 μg of pT076, 2 μg of pZT-AAVS1-L1, and 2 μg of pZT-AAVS1-R1 via Nucleofector^®^ Technology (Lonza; Houston, TX, USA) as directed by the manufacturer’s instructions; all subsequent cell-culture and experimental procedures were performed under G418 selection (50 μg/mL, Gibco 10131035; Waltham, MA, USA) to protect against shutdown of the AAVS1-integrated transgenes. The transfected cells were plated at low density (8000 cells in a 10 cm dish) to ensure that all colonies were generated from single cells; then, the cells were cultured for ~10 days and treated with dox (5 μg/mL, Sigma, D9891-25g) for 48 h. Colonies with high levels of GFP expression were captured and purified on a CytoFLEX flow cytometer (Beckman Coulter; Brea, CA, USA) at the University of Alabama Birmingham (UAB) Flow Facility. Ten purified colonies were selected and expanded.

### 4.3. Genotype Analysis

Genomic DNA (gDNA) was extracted with the DNeasy Blood and Tissue kit as directed by the manufacturer’s instructions (Qiagen; Germantown, MD, USA), and polymerase chain reaction (PCR) was performed with LR1 and LR2 primer pairs for 5′ junctions and with RR1, RR2, and RR3 primer pairs for 3′ junctions. Primer sequences are listed below:

#### DNA PCR Primer Sequences

LR1: AAVS1-a+NEO1 (1336 bp);
LR2: AAVS1-a+NEO2 (1276 bp);
AAVS1-a: 5′-ATGCAGGGGAACGGGGAT-3′;
NEO1: 5′-GCTGTGCCCAATCGTATCCAAACAGTCT-3′;
NEO2: 5′-GCTCAATCATGATATCAGGACC-3′;
RR1: AAVS1-b+TRE3G1 (924 bp);
RR2: AAVS1-b+TRE3G2 (984 bp);
RR3: AAVS1-b+TRE3G3 (1040 bp);
AAVS1-b: 5′-TCGACTTCCCCTCTTCCGAT-3′;
TRE3G1: 5′-TCACTGATAGGGAGTAAACTC-3′;
TRE3G2: 5′-AGTAAAGTCTGCATACGTTCTC-3′;
TRE3G3: 5′-ATACGTTCTCTATCACTG-3′.

### 4.4. Generation of Dox-Inducible CCND2-Overexpressing hiPSCs

#### 4.4.1. Guide RNA (gRNA) Design

The CHOPCHOP tool (https://chopchop.cbu.uib.no/, accessed on 12 March 2021) was used to design gRNA sequences targeting the promoter region of CCND2 with a threshold of 300 bp upstream for the transcription start site and an NGG protospacer adjacent motif (PAM). Forward and reverse primers were generated for each of the top-five recommended sequences (listed below) and cloned into plasmid pLKO5.sgRNA.EFS.GFP, which had been digested with BsmBI, as directed by a previously published protocol (https://bio-protocol.org/e3348, accessed on 12 March 2021). Plasmid construction was confirmed via both DNA sequencing and PCR with the U6 validation primer and the gRNA reverse primer.

#### 4.4.2. gRNA Sequences

CCND2 ON gRNA 1F:  CACCGCCCGACCCAACTTCAAACG;
CCND2 ON gRNA 2F:  CACCGCCGCGTTTGAAGTTGGGTC;
CCND2 ON gRNA 3F:  CACCGGGGGACCGCGTTTGAAGTT;
CCND2 ON gRNA 4F:  CACCGAGCGGTGACGCAAGCTGGC;
CCND2 ON gRNA 5F:  CACCGGGGGGACCGCGTTTGAAGT.

#### 4.4.3. Lentivirus Packaging and Transduction

pLKO5.sgRNA.EFS.GFP plasmids containing the CCND2-promoter–targeting gRNA sequence were cotransfected with pMD2.G and psPAX2 plasmids into 293FT cells. Seventy-two hours later, the virus-containing supernatant was collected and incubated overnight with Lenti-X Concentrator (Clontech Laboratories, 631231; San Jose, CA, USA) at 4 °C; then, the virus was precipitated by centrifugation for 1 h at 1500× *g* and 4 °C, resuspended in phosphate-buffered saline (PBS), and stored at −80 °C until use. The virus was transduced into dox-inducible dCas9-VPR hiPSCs for 48 h at a multiplicity of infection (MOI) of 20.

### 4.5. Western Blotting

Western blotting was performed as described previously [21,22]. Briefly, 1 × 107 cells were homogenized in 1 mL of RIPA lysis buffer (50 mM Tris-HCl pH 8.0, 1 mM EDTA, 1% Triton X-100, 0.1% SDS, 150 mM NaCl) containing a protease inhibitor (Sigma, 4693132001; Burlington, MA, USA) and phosphatase inhibitor (Sigma, 4906837001); then, the lysates were incubated with agitation for 1 h at 4 °C and centrifuged for 15 min at 13,000× *g* and 4 °C. Supernatants were collected, and the protein concentration was measured by a bicinchoninic acid (BCA) assay (Pierce, 23227; Rockford, IL, USA). For immunoblotting, proteins were denatured by heating for 10 min at 95 °C, separated by sodium dodecyl–sulfate polyacrylamide gel electrophoresis (SDS-PAGE), and then transferred onto a polyvinylidene difluoride (PVDF) membrane (Bio-Rad; Hercules, CA, USA). The membrane was blocked with 5% non-fat milk in tris-buffered saline with 0.1% Tween-20 (TBST) for 1 h and then incubated with primary antibodies (rtTA: Takara, San Jose, CA, USA, catalog number 631132; Cyclin D2: Cell Signaling, Danvers, MA, USA, catalog number 3741; Cas9: Cell Signaling, catalog number 14697; β-tubulin: Cell Signaling, catalog number 2128) in TBST-containing 3% bovine serum albumin (BSA) overnight at 4 °C, washed four times (10 min/wash) with TBST, incubated with secondary antibodies for 1 h, washed four more times (10 min/wash) with TBST, and developed with Enhanced Chemiluminescence Detection Reagents (ECL, Thermo Fisher; Waltham, MA, USA). Western blot images were captured with a Bio-Rad ChemiDoc System. The dCas9, rtTA, and CCND2 protein expression levels were normalized to β-tubulin protein and expressed as percentages.

### 4.6. Pluripotentcy of ^Dox^CCND2-hiPSCs

All procedures and protocols involving animals were approved by the Institutional Animal Care and Use Committee (IACUC) of the University of Alabama, Birmingham, and performed in accordance with the Guide for the Care and Use of Laboratory Animals published by the National Institutes of Health (NIH publication No 85-23). A teratoma formation assay was employed to determine the pluripotency of DoxCCND2-hiPSCs as described previously [23]. Briefly, 2 × 106 ^Dox^CCND2-hiPSCs were injected into the flanks of NOD-SCID mice, and teratomas were explanted 1.5 months later. Teratomas were processed, embedded in paraffin, and cut into sections; then, the sections were stained with hematoxylin and eosin and viewed under an Olympus microscope to identify endodermal, mesodermal, and ectodermal cells. Furthermore, ^dox^CCND2-hiPSCs were differentiated into CMs using an established protocol as described [24,25].

### 4.7. Pluripotentcy of ^Dox^CCND2-hiPSCs

Results were presented as mean ± standard error mean (SEM), and statistical analyses were performed with SPSS (version 20.0) software. Differences among different groups were tested for significance via the non-parametric Kruskall-Wallis test. A *p*-value of <0.05 was considered significant.

## Data Availability

The original contributions presented in the study are included in the article, further inquiries can be directed to the corresponding author.
